# Catecholaminergic polymorphic ventricular tachycardia in pregnancy: a case report

**DOI:** 10.1186/s13256-020-02569-5

**Published:** 2020-12-09

**Authors:** Amy Schumer, Stephen Contag

**Affiliations:** Department of Obstetrics, Gynecology and Women’s Health, University of Minnesota, 420 Delaware Street SE, Minneapolis, MN 55455 USA

**Keywords:** Inherited arrhythmia, Cardiovascular disease in pregnancy, High-risk obstetrics

## Abstract

**Introduction:**

Catecholaminergic polymorphic ventricular tachycardia (CPVT) is a genetic disorder that can cause fatal tachyarrhythmias brought on by physical or emotional stress. There is little reported in the literature regarding management of CPVT in pregnancy much less during labor.

**Case presentation:**

A gravida 2, para 1 presented to our high-risk clinic at 15 weeks gestation with known CPVT. The Caucasian female patient had been diagnosed after experiencing a cardiac arrest following a motor vehicle accident and found to have a pathogenic cardiac ryanodine receptor mutation. An implantable cardioverter defibrillator was placed at that time. Her pregnancy was uncomplicated, and she was medically managed with metoprolol, flecainide, and verapamil. Her labor course and successful vaginal delivery were uncomplicated and involved a multidisciplinary team comprising specialists in electrophysiology, maternal fetal medicine, anesthesiology, general obstetrics, lactation, and neonatology.

**Conclusions:**

CPVT is likely underdiagnosed and, given that cardiovascular disease is a leading cause of death in pregnancy, it is important to bring further awareness to the diagnosis and management of this inherited arrhythmia syndrome in pregnancy.

## Introduction

Catecholaminergic polymorphic ventricular tachycardia (CPVT) is an inherited arrhythmia syndrome caused by autosomal dominant mutations most commonly in the cardiac ryanodine receptor (RYR2). It can lead to fatal tachyarrhythmias such as bidirectional ventricular tachycardia, polymorphic ventricular premature beats, or ventricular tachycardia brought on by the release of catecholamines such as through exercise [1]. Mutated RYR2 releases more calcium ions into the cytoplasm, resulting in elevated intracellular diastolic calcium, which drives exchange of sodium and calcium through the plasma membrane via the sodium–calcium exchanger, leading to afterdepolarizations that may trigger additional action potentials or arrhythmias. Patients may present with sudden cardiac arrest or syncope. Patients have structurally normal hearts and a normal electrocardiogram making its diagnosis elusive. The prevalence is estimated to be 1:10,000 though it is likely underrecognized [1]. Sixty percent of patients with CPVT have an *RyR2* (cardiac ryanodine receptor) or* CASQ2* (cardiac calsequestrin) mutation. Mutations in *KCNJ2*, a locus on chromosome *7p1422-p22*, *Ank2*, and *TRDN* may also cause CPVT or similar tachyarrhythmia syndromes [1]. Untreated patients are at increased risk of syncope and sudden cardiac death.

CPVT can be diagnosed on an exercise stress test or on genetic testing following a cardiac arrest. While diagnosis may occur later in life, it mostly affects people less than 40 years old. Treatment typically involves a non-selective beta blocker, as well as placement of an implantable cardioverter defibrillator (ICD) if the patient has had a prior cardiac arrest or is at high risk for cardiac arrest despite maximal medical management. Additional treatments may include flecainide or left cardiac sympathetic denervation (LCSD).

There is little reported in the literature regarding management of CPVT in pregnancy much less during labor. Given the rarity of this condition and the relative infrequency of major cardiac events, little remains known about how pregnancy impacts this condition and how best to manage their labor, a time of physical exertion and stress.

## Case presentation

Our Caucasian female patient was a 28-year-old G2P1000 who presented to the maternal fetal medicine clinic for consultation with diagnosed CPVT.

She had a history of an uncomplicated first pregnancy and vaginal delivery without notable cardiac events. Two years later, she suffered a cardiac arrest during a car accident in her early twenties. During the workup following this event she was found to have a de novo *RyR2* receptor mutation diagnostic of CPVT. A single-chamber, single-coil Boston Scientific Energen™ ICD was placed. Her ICD was set to pace at less than 35 beats per minute (bpm) and discharge at greater than 240 bpm. While undergoing her evaluation, her 2-year-old child died of a sudden cardiac arrest and was presumed to carry the same mutation. Our patient’s medical history was also notable for 0.5 pack per day tobacco use. She had no known family history of cardiac disease.

During this second pregnancy she was managed by maternal fetal medicine with consults to anesthesiology, electrophysiology, cardiology, and pediatric cardiology for planning and preparation for delivery. Her second pregnancy was also complicated by a marginal cord insertion. She had no ICD discharges. During this pregnancy she was noted to have had at least nine events of non-sustained ventricular tachycardia on device interrogation, however no symptomatic episodes or syncopal events. Her cardiologist continued her prior to pregnancy flecainide, metoprolol, and verapamil during the entire pregnancy. She tolerated light exercise including walking and yoga without complication.

She presented to labor and delivery at 39 weeks for scheduled labor induction. Her vital signs were within normal limits. Her physical exam was unremarkable with regular cardiac rate and rhythm noted on auscultation and no lower extremity edema. Admission laboratory test results were notable for a hemglobin level of 12.2 g/dL, platelet count of 140 × 10^9^, and potassium, magnesium, and ionized calcium within normal limits. Her cervix was noted to be 2 cm/50% effaced/−3 station. She was placed on continuous telemetry and her ICD was interrogated. Magnesium was replaced to maintain a serum level greater than 2.0 mg/dL. A cervical balloon and intravenous administration of oxytocin were used to start the labor induction. A dexmedetomidine drip was slowly titrated for an hour preceding epidural placement, to minimize norepinephrine release. An epidural catheter was placed early in labor to minimize a stress response to labor pain. Bupivacaine without epinephrine was used in the epidural to minimize arrhythmias. Under the supervision of an electrophysiologist an esmolol drip was initiated during labor induction, given an increased frequency of premature ventricular contractions (PVCs). The electrophysiologist was on the floor or immediately available at all times during her labor induction to manage her cardiac medications and her ICD settings at all times. Her ICD pacing rate was increased from 35 to 80 bpm to decrease the frequency of PVCs. She subsequently underwent artificial rupture of membranes at 4 cm of cervical dilation. Her labor progressed quickly, and she was completely dilated less than 2 hours later. She pushed effectively over less than 15 minutes and delivered a liveborn female infant weighing 2630 g and with Apgar of 8 and 9 at 1 and 5 minutes, respectively. Quantitative blood loss was 321 mL. A discharge hemoglobin level was 9.2 g/dL. Her epidural was discontinued at 2 hours after delivery.

Early on postpartum day 1 she was noted to have larger than expected blood loss likely secondary to uterine atony. Intravenous oxytocin infusion was continued. A spinal block was placed as her epidural had been removed in the immediate postpartum period. Once adequate pain coverage was achieved, a manual uterine sweep was performed for an additional 1900 mL quantitative blood loss, in order to improve uterine tone and prevent further blood loss. Her vital signs remained stable and her hemoglobin nadir was 9.2. She received antibiotic prophylaxis. Later on postpartum day 1 she was transitioned back to orally administered flecainide, orally administered metoprolol, and her ICD settings were adjusted with the pacing rate returned to 35 bpm. Her esmolol drip was slowly weaned, and telemetry was continued for 24 hours postpartum without incident. The following 6-month postpartum period was uncomplicated and she continued on flecainide, verapamil, and metoprolol under the supervision of her cardiologist. She opted to use condoms for contraception. Her infant underwent genetic testing for her *RyR2* mutation and was found to be negative.

## Discussion

This case demonstrates the successful pregnancy and labor management of a woman with CPVT. Despite the severity and frequency of this woman’s condition, careful management allowed her to have a successful vaginal birth with a good outcome. The case reports discussed below emphasize the need for a multidisciplinary team approach with a clear understanding of the pathophysiology as well as of the implementation of a carefully designed management algorithm to care for the patient antepartum as well as intrapartum and postpartum.

Our clinical management followed standard recommendations for prevention of arrhythmias among individuals with CPVT. Recent publications have focused on the frequency of events among pregnant women compared with non-pregnant women which does not appear to be increased as documented in a recent review by Cheung* et al.* [2]. The risk of ventricular events in pregnancy is unknown. Two case reports of CPVT document ICD shocks in pregnancy: one patient undergoing three shocks for appropriate arrhythmias in the first trimester [3]. The other patient underwent a single ICD shock at 26 weeks gestation in the setting of medication non-compliance and subsequently delivered a preterm infant at 30 weeks gestation as a result of preterm prelabor rupture of membranes [4]. A larger retrospective review of 228 pregnancies showed no increased risk of events during or after pregnancy; however, the study was limited by a small patient population further confounded by relatively rare rates of cardiac events. Six patients, none of which were taking a beta blocker at the time, had a cardiac event during pregnancy or in the postpartum period. Though not significant, this study suggests the importance of continued medication compliance throughout pregnancy.

One case reported in the literature emphasized the multidisciplinary approach with adequate planning of care used in managing a woman who presented at 15 weeks of gestation and ultimately delivered by cesarean birth for fetal indication [5]. This woman did not meet criteria for implantable defibrillator placement and was able to exercise daily. She was treated with nadolol 1 mg/kg a day. Similar to our case, she declined prenatal diagnosis. In this report, simulations for management of CPVT were performed with the staff from obstetrics, anesthesia, and cardiology prior to the delivery. A plan for telemetry was in place during labor and no episodes of intrapartum maternal arrhythmias were recorded. This contrasts with our patient who had an implanted ICD, and presented with recurrent episodes of arrhythmia throughout pregnancy that increased during the labor process but were successfully managed with the implementation of a carefully designed management protocol [5]. An earlier case report involved a young woman managed with an ICD and propranolol and propafenone [4]. She had two episodes of admission for maternal arrhythmia secondary to non-adherence with medications. She was induced at 30 weeks after development of prolonged rupture of membranes (PROM). During the labor process she continued her therapeutic regimen, had cardiac telemetry, and received an early epidural for pain management. She had a vaginal delivery of a preterm infant which tested negative for the RYR2 mutation [4].

There are earlier case reports that focus on various aspects of management such as use of anesthetics during labor and delivery [6]. A third case report by Walker* et al.* [7] discusses management of bidirectional ventricular tachycardia secondary to presumed CPVT. In this case urgent management in labor and delivery included use of flecainide for rhythm and rate control followed by emergency cesarean delivery.

Cardiovascular disease is a leading cause of death in pregnancy and the postpartum period accounting for 4.23 deaths per 100,000 live births [8]. In other words, 26.5% of US pregnancy-related deaths are attributed to cardiovascular disease [8]. While many cardiovascular deaths can be attributed to structural heart disease, 60% are due to arrhythmias with a structurally normal heart [2, 8]. CPVT is estimated to affect 1 in 10,000 people; however, it is likely underrecognized. Only one-third of patients have a family history of syncope or sudden cardiac death [9]. It is estimated that in untreated patients with CPVT, mortality rates approach 30–50% by age 40 [9].

CPVT is diagnosed in patients with a structurally normal heart, normal ECG, and bidirectional ventricular tachycardia or polymorphic ventricular premature beats provoked by exercise or stress typically in patients less than 40 years old. Diagnosis can be confirmed with genetic testing. CPVT is known to have incomplete penetrance. Once an index family member has been diagnosed, other family members can be diagnosed on the basis of the presence of PVCs or polymorphic ventricular tachycardia. Once diagnosed, patients are recommended to limit strenuous sports and activities. All symptomatic patients and asymptomatic patients found to carry the gene mutation should be placed on a beta blocker. An ICD is recommended if the patient has had a cardiac arrest or continues to have tachyarrhythmias despite optimal medical management [1]: in cases of recurrent syncope, continued arrhythmias, or ICD shocks while on a beta blocker, or if a beta blocker is contraindicated, consider LCSD [1].

In pregnancy, CPVT can be diagnosed on a submaximal exercise stress test eliciting 80% of the predicted maximal heart rate [8, 10]. Beta blockers are generally safe in pregnancy with the exception of atenolol. Use of atenolol is controversial as it has been shown to cross the placenta and is associated with small for gestational age, bradycardia, and hypoglycemia [11]. Non-selective beta blockers such as propranolol are preferred. Non-selective beta blockers may impact uterine contractions and peripheral vasodilation and therefore there is a small association with lower birth weight, preterm labor, neonatal hypoglycemia, and hyperbilirubinemia [10]. Selective beta blockers like metoprolol have less theoretical complications in pregnancy but may be less effective. Labetalol has not been shown to cause any adverse effects in pregnancy at therapeutic doses [8]. As a second-line agent, flecainide may be used. Flecainide is a sodium channel blocker that has been shown to be teratogenic, with limited data in humans demonstrating its safety [8, 10]. If it is required to prevent PVCs, it is recommended to be stopped around time of conception and restarted after the first trimester. An ICD may be placed in pregnancy whenever indicated. After 8 weeks gestation, a single-chamber device is recommended [10]. LCSD should be delayed until after pregnancy [10].

Despite a dearth of data, pregnancy and labor are believed to place women at higher risk of tachyarrhythmias. This is felt to be the result of significant physiologic changes including ventricular remodeling, increased stroke volume and plasma volume, which lead to increased cardiac output; and a decrease in vascular resistance [12]. Cardiac output continues to increase throughout pregnancy and during labor [12]. This is accompanied by increases in heart rate and blood pressure during labor. As a result, pregnancy and labor are considered arrhythmogenic. Continuation of medications in labor is essential. Even a single missed dose can lead to a tachyarrhythmia [10]. Intravenous esmolol administration has been used owing to its short half-life and can be rapidly titrated. Patients should be kept below their arrhythmic window, which is the heart rate when PVCs occur. This is often around 100–110 bpm [10]. There is no evidence to suggest that there are improved outcomes with cesarean delivery over vaginal birth [13]. Adequate pain control, however, is important. In labor, an early epidural is recommended in order to minimize further catecholaminergic stimulus secondary to pain [14]. It is recommended that a multidisciplinary team comprising a labor and delivery nurse, cardiac nurse, anesthesiology, obstetrician, and cardiologist with knowledge of tachyarrhythmias be readily available [15]. Intrapartum continuous cardiac monitoring should be considered [10]. Additionally, an external cardioverter defibrillator device should be available on the unit. Consider an arterial line for highest-risk patients [10].

Postpartum there are further physiological changes in heart rate, with increasing vascular resistance and plasma volume [8]. We recommended that the patient follow up with her cardiologist within 1–2 weeks to evaluate cardiac function, and if the ICD is present, interrogate for evidence of PVC, and to adjust medications [8]. We also recommend that the newborn should undergo genetic testing prior to discharge if a mutation has been identified in the mother.

## Conclusions

Pregnancy and labor are believed to place women at higher risk of tachyarrhythmias. The risk of ventricular events in pregnancy, however, remains unknown given the paucity of cases in the literature. Management of CPVT in pregnancy requires a multidisciplinary team. Implanted ICD and antiarrhythmics, especially beta blockers, remain essential to management of this condition. CPVT is likely underdiagnosed and, given that cardiovascular disease is a leading cause of death in pregnancy, it is important to bring further awareness to the diagnosis and management of this inherited arrhythmia syndrome in pregnancy.

## Data Availability

Not applicable

## Abbreviations

Bpm: Beats per minute
*CASQ*: Cardiac calsequestrin
CPVT: Catecholaminergic polymorphic ventricular tachycardia
ICD: Implantable cardioverter defibrillator
LCSD: Left cardiac sympathetic denervation
PVC: Premature ventricular contraction
RN: Registered nurse
*RyR2*: Cardiac ryanodine receptor
